# Prooxidation and Cytotoxicity of Selenium Nanoparticles at Nonlethal Level in Sprague-Dawley Rats and Buffalo Rat Liver Cells

**DOI:** 10.1155/2020/7680276

**Published:** 2020-08-14

**Authors:** Haidong Wang, Yudan He, Lujie Liu, Wenjing Tao, Geng Wang, Wanjing Sun, Xun Pei, Zhiping Xiao, Yuyue Jin, Minqi Wang

**Affiliations:** ^1^Key Laboratory of Molecular Animal Nutrition, Ministry of Education, College of Animal Science, Zhejiang University, Hangzhou 310058, China; ^2^Department of Animal Science, Jiangxi Biotech Vocational College, 608 Nanlian Road, Nanchang, 330200 Jiangxi, China

## Abstract

The effects of selenium nanoparticles (SeNPs) on the antioxidant capacity in Sprague-Dawley (SD) rats were investigated. The rats were given intragastric administration of an SeNP suspension at doses of 0, 2, 4, and 8 mg Se/kg BW for two weeks. The antioxidant capacity in serum and organic tissues (liver, heart, and kidney) and the gene expression levels of glutathione peroxidase 1 (GPX1) and glutathione peroxidase 4 (GPX4) in the liver were measured. Buffalo rat liver (BRL) cell lines were further constructed to explore the cytotoxicity mechanism induced by SeNPs through the determination of antioxidant capacity; cell activity; apoptosis; and Caspase-3, Caspase-8, and Caspase-9 family activities. The results showed that SeNP administration over 4.0 mg Se/kg BW decreased the antioxidant capacities in the serum, liver, and heart and downregulated mRNA expression of GPX1 and GPX4 in the liver. The BRL cell line experiments showed that treatment with over 24 *μ*M SeNPs decreased the viability of the cells and damaged the antioxidant capacity. Flow cytometry analysis showed that decreased cell viability induced by SeNPs is mainly due to apoptosis, rather than cell necrosis. Caspase-3 and Caspase-8 activities were also increased when BRL cells were treated with 24 *μ*M and 48 *μ*M SeNPs. Taken together, a nonlethal level of SeNPs could impair the antioxidant capacity in serum and organic tissues of rats, and the liver is the most sensitive to the toxicity of SeNPs. A pharmacological dose of SeNPs could lead to cytotoxicity and induce cell death through apoptosis and extrinsic pathways contributing to SeNP-induced apoptosis in BRL cells.

## 1. Introduction

Selenium (Se) is an essential trace mineral for human beings and animals, and its deficiency is the cause for susceptibility to various diseases, such as Keshan disease, Kashin-Beck disease, atherosclerosis, and cancer [1–3]. Selenium and selenocompounds play a crucial role in the oxidation-reduction system. Selenium supplementation can improve oxidation enzyme activity, reduce reactive oxygen species (ROS), and relieve cellular damage [4–6]. Selenium commonly occurs as selenate (Se^+6^), selenite (Se^+4^), selenide (Se^−2^), and elemental selenium (Se^0^) [7]. Both the efficacy and toxicity of Se compounds are strictly dependent on its concentration and chemical forms as well as redox potential.

Various studies showed that Se nanoparticles (SeNPs) have higher bioavailability and lower toxicity compared to organic and inorganic Se [8–11]. Due to the vulnerability of cancer cells to oxidative stress, the idea of targeting the antioxidant capacity of tumor cells has been considered as a promising therapeutic strategy [12]. At nonlethal doses, Se typically turns into a prooxidant with growth-inhibiting properties and with high cytotoxic activities [13]. SeNPs were reported to selectively inhibit the growth of tumor with no side effect on normal cells [14–16]. Therefore, the SeNPs, as a promising material for biomedical applications, especially for cancer treatment, have attracted more attention. However, as Se has an extremely narrow threshold range between beneficial biological dosage and toxicity limit, a pharmacological level of SeNPs may also have potential toxicity and ill effects [17–20]. Besides, oxidation promotion is considered to be the main disadvantage for nanoparticles, and it gets increasing attention, which has been the bottleneck for SeNP application [21].

To the best of our knowledge, very limited work has been conducted to ascertain the effects of SeNPs at the nonlethal level on antioxidant capacity in cells and animals, and its toxicity to normal cells. To clarify and characterize the safety profile of SeNPs, we investigated the antioxidant capacity in rats and buffalo rat liver (BRL) cells and the cytotoxicity to SeNP exposure. The results obtained will provide a theoretical basis for nano-Se-based drug development as chemotherapeutic agents in the future.

## 2. Materials and Methods

### 2.1. Preparation and Characterization of SeNPs

The SeNPs were prepared following the method by He et al. with some modifications [7]. Briefly, 100 mL chitosan solution (8 mg/mL) was prepared by dissolving chitosan in deionized water under stirring overnight at 25°C. One mL sodium selenite solution (125 mM) and TPP solution (1%, *w/w*) were slowly added into the mixtures, respectively, under magnetic stirring overnight, then 5 mL ascorbic acid solution (100 mM) was added into the mixture, and reconstituted to a final volume of 25 mL with the concentration of 5 mM Se. The solutions were dialyzed against Milli-Q water until no Se was detected in the outer solutions, which was determined by inductively coupled plasma-atomic emission spectroscopy (ICP-AES) analysis. Then, solutions were serially filtered through 0.22 *μ*m pore-size filters to get rid of bacteria. The Se concentration of the obtained SeNP solutions was also determined by ICP-AES analysis and stored at 4°C for subsequent experiments.

The average diameter, size distribution, and surface charges of the nanoparticles were measured by the Zetasizer Nano ZS particle analyzer (Zetasizer Nano ZS90, Malvern, UK). The morphologies and particle sizes of the samples were analyzed using transmission electron microscopy (TEM, JEM-100SX, JEOL, Japan).

**Figure 1:**
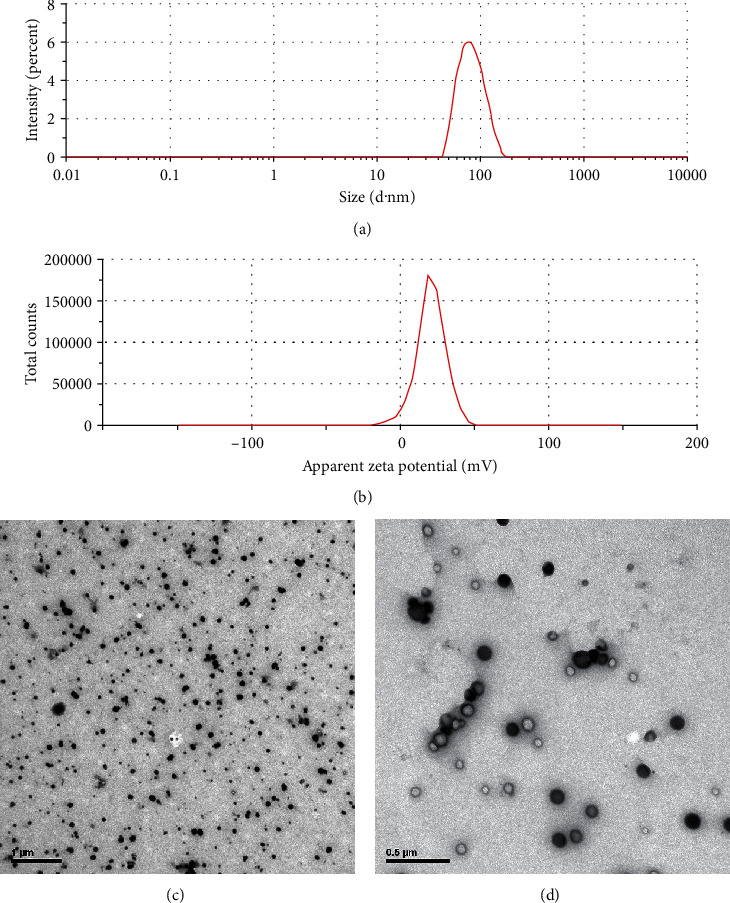
Characterization of SeNPs: (a) size distribution and (b) zeta potential distribution of SeNPs; (c and d) TEM images of SeNPs. Scale bar = 1 *μ*m and 0.5 *μ*m, respectively.

As shown in Figure 1, SeNPs were characterized with an average diameter of 79.88 nm (Figure 1(a)) and a mean zeta potential of +29 mV (Figure 1(b)). The TEM analysis revealed that SeNPs were spherical and well dispersed particles (Figures 1(c) and 1(d)).

### 2.2. Animals and Experimental Treatment

All animal experiments were approved by the Zhejiang University Animal Research Ethics Board and were done under the guidelines of the China Council for Animal Care.

Six-week-old male Sprague-Dawley (SD) rats and standard ration (0.08 mg Se/kg diet) were supplied by the Experimental Animals Center of Zhejiang University. After a one-week adaptation period fed with standard ration, a total of 40 rats were assigned randomly into four treatments, with ten mice in each treatment. The rats were intragastrically administered saline in the control treatment, while rats in other treatments were intragastrically administered SeNPs with doses of 2.0, 4.0, or 8.0 mg Se/kg BW, which was 25, 50, and 100 times the Se level in the standard ratio (0.08 mg Se/kg) for rats, correspondingly. Each rat received intragastric administration once a day at 10 a.m. for 14 consecutive days. The cages were placed in an air-conditioned room (temperature: 22 ± 3°C; relative humidity: 50 ± 10%) with a 12 h light/dark cycle. All rats were allowed free access to food and water, and their activity was carefully observed and recorded every day. After the gavage experiment, the rats were fasted overnight and then euthanized with pentobarbital sodium. The blood samples were collected via the aortaventralis into a 5 mL evacuated tube, then centrifuged at 3000 g for 10 min to isolate serum and stored at -30°C. The rats were executed by spinal dislocation, and tissue samples of liver, kidney, and heart were collected and weighed, then stored at -80°C for further analysis.

### 2.3. Serum and Tissue Antioxidant Capacity Measurement

The samples from the liver, kidney, and heart were homogenized in ice-cold saline and centrifuged at 4000 rpm at 4°C for 10 minutes. The obtained tissue supernatants and serum samples were used to determine the antioxidant capacity.

The protein concentration was determined by the bicinchoninic acid assay (BCA assay). The activity of glutathione peroxidase (GSH-Px) was measured using the assay kit based on the principle that oxidation of glutathione (GSH) and H_2_O_2_ could be catalyzed by GSH-Px to produce oxidized glutathione (GSSG) and H_2_O [22]. The activity of superoxide dismutase (SOD) was determined according to the xanthine oxidase method and using the water-soluble tetrazolium salt (WST-1) method [23]. Thioredoxin reductase (TrxR) activity was determined by a commercial TrxR kit based on the principle that TrxR catalyzed the reduction of 5,5′-dithiobis (2-nitrobenzoic acid) (DTNB) by NADPH to produce 5-thio-2-nitrobenzoate (TNB) and NADP^+^. TrxR activity could be calculated by measuring the increased rate of TNB at a 412 nm wavelength [24]. Catalase (CAT) activity was evaluated by analyzing the rate of CAT to decompose H_2_O_2_ and measured at a 405 nm wavelength by using molybdenic acid [25]. The spectrometric method was applied to assess total antioxidant capacity (T-AOC). In the reaction mixture, ferric ion was reduced by antioxidant reducing agents and the blue complex Fe^2+^-TPTZ (2,4,6-tri(2-pyridyl)-s-triazine) was produced. The concentrations of malondialdehyde (MDA) were analyzed using the thiobarbituric acid (TBA) method [26]. The abilities of antisuperoxide anion (ASA) was measured using the xanthine-xanthine oxidase system and detected at a 550 nm wavelength using the Griess reagent. Inhibition of hydroxyl radical (IHR) was measured according to the Fenton system and detected at a 550 nm wavelength using the Griess Reagent [27]. Total glutathione (T-GSH) and GSSG were measured by using the cyclic reaction of DTNB [28]. The activity of GSH was evaluated by using the DTNB technique [29]. The content of H_2_O_2_ was assessed using the molybdenic acid method.The commercial assay kits for GSH-Px, SOD, TrxR, CAT, T-AOC, MDA, ASA, IHR, T-GSH, GSSG, GSH, and H_2_O_2_ were purchased from Nanjing Jiancheng Bioengineering Institute (Nanjing, Jiangsu, China).

### 2.4. RNA Isolation and Quantitative Real-Time PCR (qPCR) Assay

Total RNA was extracted using the TRIzol Reagent (Invitrogen, Carlsbad, CA, USA) according to the manufacturer's procedures. Complementary DNA (cDNA) was synthesized from 2 *μ*g of total RNA using the First Strand cDNA Synthesis Kit (Toyobo, Osaka, Japan) following the manufacturer's protocol. qPCR analyses were performed with the SYBR Green Master Mix (Takara, Dalian, China). qPCR application was performed using a CFX96™ qPCR system (Bio-Rad, Hercules, CA, USA) in triplicate. The following amplification protocol was used: 95°C for 1 min, followed by 40 cycles (95°C for 15 s, 58°C for 20 s, and 72°C for 20 s). *β*-Actin was used as a housekeeping gene to normalize target gene levels. Primer sequences for the target genes (GPX1 and GPX4) were synthesized by Invitrogen (Shanghai, China) and listed in Table 1. Relative expression levels of the target genes were calculated using the 2^−ΔΔCt^ method [30].

### 2.5. Transmission Electron Microscopy (TEM) Observation

A cubic sample of about 1 mm^3^ was removed from the liver. The liver tissue sample was fixed in 2.5% glutaraldehyde and cacodylate solution (0.1 M/dm^3^, pH 7.2-7.4) at 4°C and washed by cacodylate solution (0.1 M/dm^3^) for three times. After that, the sample was fixed in 1% osmic acid for 1 h and washed by cacodylate solution (0.1 M/dm^3^) thrice before dehydrating with gradient concentration acetone (30%, 50%, 70%, 80%, 90%, and 100%). The tissue was finally embedded in epoxy resin (Epon 812). The embedded samples were sectioned (40 to 50 nm) and stained by lead citrate solution and uranyl acetate followed by TEM observation. The morphology was observed by TEM (JEOL, JEM-2100F), working on an acceleration voltage of 200 kV.

### 2.6. Cell Culture and Treatment

The BRL cell lines were purchased from the Institute of Biochemistry and Cell Biology, China Academy of Sciences (Shanghai, China). The BRL cells were cultured in Dulbecco's modified Eagle's medium (DMEM) supplemented with 10% fetal bovine serum (FBS), 100 U/mL penicillin, and 100 *μ*g/mL streptomycin (Gibco, Carlsbad, CA, USA) in the CO_2_ incubator (5% CO_2_, 37°C, and 95% relative humidity). Cells were passaged regularly using 0.25% trypsin (0.1% EDTA) and subcultured to 80% confluence before the cell experiment. SeNPs were prepared and confirmed with the former protocol. Cells were cultured with the medium containing different concentrations of SeNPs (0, 0.1, 0.5, 1, 12, 24, and 48 *μ*M) in a CO_2_ incubator (5% CO_2_, 37°C, and 95% relative humidity) for 24 h.

### 2.7. Cell Viability Assay

Cell viability was determined using the MTT Assay Kit (Gibco, Carlsbad, CA, USA) according to the manufacturer's instructions. Cells at the exponential growth stage were seeded into 96-well plates with a density of 1 × 10^4^/well and incubated at 37°C with 5% CO_2_ for 12 h. Then, the medium was removed, and cells were treated with different concentrations of SeNPs. After that, 10 *μ*L MTT reagent was added into each well and incubated at 37°C with 5% CO_2_ for 4 h. After the medium was removed, cells were suspended in 150 *μ*L dimethylsulfoxide (DMSO) for 15 min. At the end of the experiments, the optical density (OD) was measured using SpectraMax M5 (Molecular Devices, California, USA) at a 570 nm wavelength. Cell viability (%) was calculated as follows: (OD_treatment_/OD_control_) × 100%.

### 2.8. Cell Antioxidant Capacity

Cells were seeded in 6-well plates. After SeNP treatment as described in Section 2.6, cells were harvested and washed twice with cold phosphate-buffered saline (PBS). Cells were lysed in cold lysis buffer (1% Triton X-100) on ice for 30 min. During this period, each tube was gently shaken vortically four times, each time for 30 s. Next, the cells were centrifuged at 10000 rpm for 1 min at 4°C. The supernatants were collected carefully. The levels of GSH-Px, SOD, CAT, MDA, and T-AOC were analyzed by using a commercial kit (Nanjing Jiancheng Bioengineering Institute, Nanjing, China) according to the principle of the oxidation of GSH and H_2_O_2_ via GSH-Px [22], the WST-1 method [23], the molybdenic acid method [25], the TBA technique [26], and the spectrometric method, respectively.

### 2.9. Determination of Cell Apoptosis Rate

To detect the apoptosis of BRL cells treated with SeNPs, an Annexin V-FITC/PI apoptosis detection kit (Tianjin Sungene Biotech Co., Ltd., Tianjin, China) was used according to the manufacturer's instruction. The BRL cells were seeded in 6-well plates. After incubation with different concentrations of SeNPs, cells were collected into 10 mL centrifugal tubes and washed twice with cold PBS, then centrifuged for 5 min at 1000 rpm at 4°C. Cells were suspended with 1 × binding buffer at a concentration of 1 × 10^6^ cells/mL in 10 mL centrifugal tubes. Next, 100 *μ*L of resuspended cells was added into new 10 mL centrifugal tubes. Then, 5 *μ*L Annexin V-FITC was added into the tubes and incubated at room temperature in the dark for 10 min. Then, 5 *μ*L of propidium iodide (PI) was added into the tubes and incubated at room temperature in the dark for 5 min. Finally, PBS was added to 500 *μ*L. The samples were immediately analyzed by a flow cytometer (BD Biosciences, San Jose, CA, USA) with an excitation wavelength of 488 nm in 1 h.

### 2.10. Cell Caspase Activity Assay

Caspase-3, Caspase-8, and Caspase-9 activities in BRL cells were measured with a caspase activity assay kit (Nanjing Jiancheng Bioengineering Institute, Nanjing, China) according to the manufacturer's instructions. Briefly, cells were seeded in 6-well plates and treated with SeNPs. Cells were harvested, then washed twice by cold PBS and lysed in cold lysis buffer (1% Triton X-100) on ice for 30 min, followed by centrifugation at 14000 g for 10 min at 4°C. After that, the supernatants were collected and immediately measured for protein concentration with the protein assay kit. The supernatants, reaction buffer, and specific caspase substrates (Ac-DEVD-*p*NA for Caspase-3, Ac-IETD-*p*NA for Caspase-8, and Ac-LEHD-*p*NA for Caspase-9) were added into 96-well plates and incubated at 37°C for 2 h. OD was measured at a 405 nm wavelength using SpectraMax M5 (Molecular Devices, California, USA). Caspase activities were calculated by the OD_treatment_/OD_control_ ratio.

### 2.11. Statistical Analysis

Data are presented as the mean ± standard deviation (SD). Differences between the mean values were assessed with one-way analysis of variance (ANOVA) followed by Duncan's multiple range test for multiple comparisons. *P* values < 0.05 were considered as statistically significant. All statistical analyses were performed using SPSS version 17.0 for Windows (SPSS Inc., Chicago, IL, USA).

## 3. Results

### 3.1. Serum Antioxidant Capacity in Rats

As shown in Table 2, SeNPs administered at a dose of 8 mg Se/kg BW decreased the levels of T-AOC, GSH, and ASA and increased the levels of MDA, CAT, GSSH, and H_2_O_2_ (*P* < 0.05). The levels of CAT, GSSH, and H_2_O_2_ were increased by SeNPs at a dose of 4 mg Se/kg BW (*P* < 0.05), while the levels of other biomarkers exhibited no significant differences from the control treatment. The results indicated that SeNPs administered at a dose of over 4 mg Se/kg BW could damage the serum antioxidant capacity in rats.

### 3.2. Organic Tissue Antioxidant Capacity in Rats

**Figure 2:**
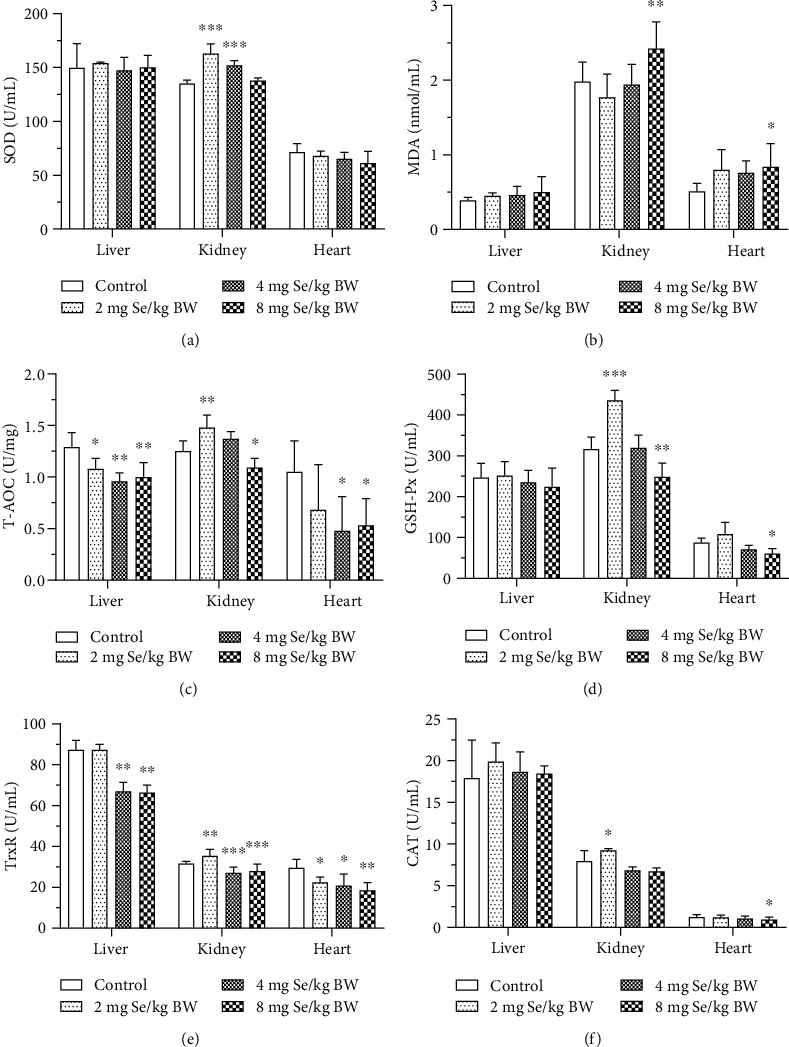
Effect of SeNPs on tissue antioxidant capacity. The levels of (a) SOD, (b) MDA, (c) T-AOC, (d) GSH-Px, (e) TrxR, and (f) CAT activities in the liver, kidney, and heart tissues. Data are expressed as the mean ± SD, *n* = 10. ^∗^, ^∗∗^, and ^∗∗∗^ indicate significant difference as compared with the control treatment (*P* < 0.05, 0.01, and 0.001 respectively). SOD: superoxide dismutase; MDA: malondialdehyde; T-AOC: total antioxidation capability; GSH-Px: glutathione peroxidase; TrxR: thioredoxin reductase; CAT: catalase.

As shown in Figures 2(a)–2(f), with the same dose of SeNP administration, the levels of biomarkers in selected organic tissues were different. SeNP administration at doses of 2 and 4 mg Se/kg BW increased SOD activity in the kidney (*P* < 0.01, Figure 2(a)); however, there are no significant differences in SOD activities in the liver and the heart (*P* > 0.05). SeNP administration at a dose of 8 mg Se/kg BW increased the level of MDA in the kidney and the heart (*P* < 0.05), while no significant difference on MDA level was found in the liver (*P* > 0.05; Figure 2(b)). SeNP administration at a dose of 2 mg Se/kg BW decreased liver T-AOC (*P* < 0.01), while kidney T-AOC was increased (*P* < 0.01). T-AOC in all three selected organic tissues were decreased by SeNP administration at a dose of 8 mg Se/kg BW (*P* < 0.05, Figure 2(c)). SeNPs did not affect GSH-Px activity in the liver (*P* > 0.05). Nevertheless, 2 mg Se/kg BW SeNP administration increased GSH-Px activity in the kidney (*P* < 0.05), and 8 mg Se/kg BW SeNPs decreased GSH-Px activity in kidney and the heart (*P* < 0.05, Figure 2(d)). SeNPs at 4 and 8 mg Se/kg BW decreased TrxR activities in all three selected organic tissues (*P* < 0.05). SeNP administration at a dose of 2 mg Se/kg BW increased TrxR activity in the kidney (*P* < 0.05) but decreased TrxR activity in the heart (*P* < 0.05, Figure 2(e)). SeNPs did not affect CAT activity in the liver (*P* > 0.05). SeNP administration at a dose of 8 mg Se/kg BW decreased CAT activity in the heart (*P* < 0.05) but increased CAT active in the kidney (*P* < 0.05, Figure 2(f)). The results indicated that the kidney is the most tolerant organ to pharmacological SeNP administration, followed by the heart and the liver.

### 3.3. Liver Tissue Ultrastructure

**Figure 3:**
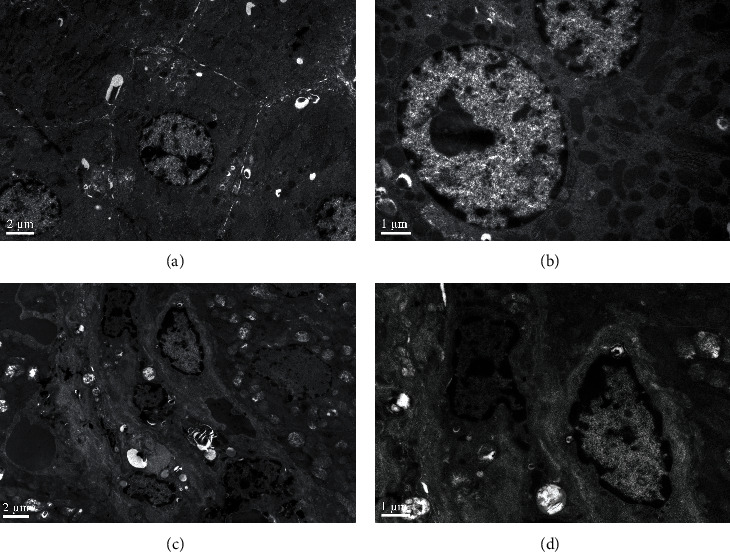
TEM photographs of liver tissue: (a and b) from the control treatment; (c and d) from 8 mg Se/kg BW SeNP treatment. Scale bar = 2 *μ*m and 1 *μ*m.

Liver tissue ultrastructure was observed by TEM and shown in Figure 3. Abundant mitochondria with an intact membrane and matrix, round nucleus with finely granular chromatin and a prominent nucleolus, and endoplasmic reticulum were found in the control treatment (Figures 3(a) and 3(b)). However, in 8 mg Se/kg BW SeNP treatment, hepatocytes lost membrane integrity and showed up swollen, and chromatin margins were gathered, which indicated that the liver cells were at the early stage of apoptosis. Meanwhile, the mitochondria were swollen, and the endoplasmic reticulum was not found (Figures 3(c) and 3(d)). These results indicated that SeNP administration at a dose of 8 mg Se/kg BW severely damaged the cell ultrastructure in the liver.

### 3.4. mRNA of GPX1 and GPX4 in the Liver

**Figure 4:**
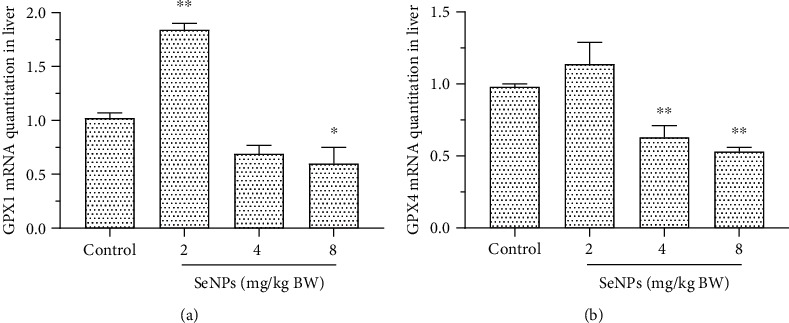
Effect of SeNPs on the mRNA expression of GPX1 (a) and GPX4 (b) in the liver. Data are expressed as the mean ± SD, *n* = 6. ^∗^ and ^∗∗^ indicate significant difference as compared with the control treatment (*P* < 0.05 and 0.01, respectively). GPX1: glutathione peroxidase 1; GPX4: glutathione peroxidase 4.

As shown in Figures 4(a) and 4(b), SeNP administration at a dose of 8 mg Se/kg BW significantly decreased GPX1 mRNA level in the liver (*P* < 0.05), while 2 mg Se/kg BW SeNPs significantly increased GPX1 mRNA level in the liver (*P* < 0.01). Four and 8 mg Se/kg BW SeNPs significantly decreased GPX4 mRNA level in the liver (*P* < 0.01).

### 3.5. Cell Viability in BRL Cells

**Figure 5:**
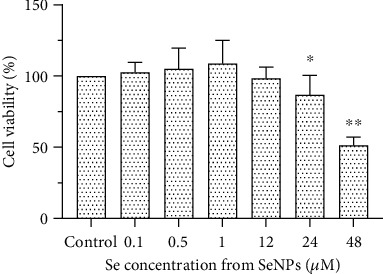
Effect of SeNPs on cell viability of BRL cells determined by MTT assay. Cell viability of BRL cells treated with different concentrations (ranging from 0.1 to 48 *μ*M) of SeNPs for 24 h. Data are expressed as the mean ± SD, *n* = 8. ^∗^ and ^∗∗^ indicate significant difference as compared with the control treatment (*P* < 0.05 and 0.01, respectively).

As shown in Figure 5, cell viability was decreased significantly (*P* < 0.05) when cells were treated with SeNPs at concentrations of 24 *μ*M and 48 *μ*M. However, SeNPs at doses of 0.1, 0.5, 1, and 12 *μ*M exhibited no growth inhibition on BRL cell lines (*P* > 0.05).

### 3.6. Cell Antioxidant Capacity in BRL Cells

As shown in Table 3, SeNPs at a concentration of 48 *μ*M significantly decreased the level of SOD, T-AOC, and GSH-Px in BRL cells (*P* < 0.01), while the level of MDA and CAT were increased (*P* < 0.001). SeNPs at a concentration of 24 *μ*M decreased the levels of T-AOC and GSH-Px (*P* < 0.05), while the levels of SOD, MDA, and CAT were increased (*P* < 0.001). SeNPs at concentrations of 1 and 12 *μ*M increased the levels of SOD and T-AOC (*P* < 0.05). SeNPs at concentrations of 0.1, 0.5, and 1 *μ*M increased GSH-Px activity (*P* < 0.01). The results indicated that the intracellular antioxidant capacity was damaged when SeNPs were administered over 24 *μ*M.

### 3.7. Apoptosis in BRL Cells

**Figure 6:**
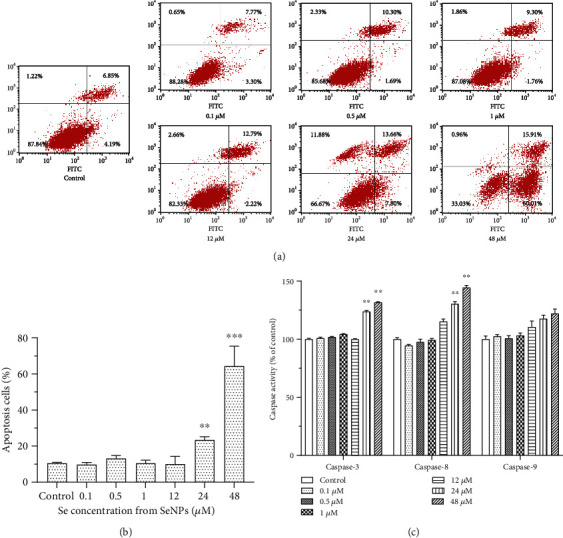
Effect of SeNPs on cell apoptosis and caspase activities: (a) SeNP-induced apoptosis in BRL cells by flow cytometric analysis. Data presented are representative of flow cytometric experiments conducted in triplicate. (b) The apoptosis rate of BRL cells treated with different concentrations (ranging from 0.1 to 48 *μ*M) of SeNPs for 24 h. (c) Analysis of caspase family activities (% of control) in SeNP-induced apoptosis in BRL cells. Data are expressed as the mean ± SD, *n* = 5. ^∗^, ^∗∗^, and ^∗∗∗^ indicate significant difference as compared with the control (*P* < 0.05, 0.01, and 0.001 respectively).

A double staining assay (Annexin V-FITC and PI) was used to determine whether apoptosis was involved in the SeNP-induced cell viability decrease. The results of Annexin V/PI assay (Figure 6(a)) showed that the percentages of total apoptotic cells treated with 24 *μ*M and 48 *μ*M SeNPs for 24 h were 23.38% and 64.34% vs. 10.49% for the control (*P* < 0.01). There were no differences in the treatments of 0.1, 0.5, 1, and 12 *μ*M SeNPs (*P* > 0.05) (Figure 6(b)). To identify the signaling pathways involved in SeNP-induced apoptosis, the activities of Caspase-3, Caspase-8, and Caspase-9 were measured. As shown in Figure 6(c), the activities of Caspase-3 and Caspase-8 were significantly increased under the exposure of 24 and 48 *μ*M SeNPs (*P* < 0.01), whereas no significant effect was found on Caspase-9 activity (*P* > 0.05).

## 4. Discussion

Due to lower toxicity and better biological activity, promising applications of SeNPs in life science, especially their potential use in cancer treatment and drug delivery, have attracted increasing attention [31]. However, chemopreventive (antitumorigenic) levels of Se may lead to toxicity [32]. And the toxicity of pharmacological Se is mainly a result of its oxidation promoting effects [33]. Besides, oxidative stress is the primary mechanism of toxicity induced by nanoparticles [21]. These side effects of SeNPs have been the bottleneck of safe SeNP applications. Therefore, it is necessary to investigate the effect of SeNPs at the nonlethal level on the antioxidant capacity for humans and animals, to pave the way for future applied research.

GSH-Px is an important Se-containing enzyme, which catalyzes the reduction of lipid hydroperoxides and hydrogen peroxide by GSH as the hydrogen donor to water and corresponding alcohols specifically, and TrxRs can catalyze the reduction of oxidized thioredoxin (Trxs) by using NADPH as an electron donor. And TrxR can also catalyze other endogenous substrates to exert antioxidant substances, including ascorbyl radical, lipoic acid, lipid hydroperoxide, and ubiquinone [34]. In our study, SeNPs at nonlethal levels increased levels of MDA, GSSH, CAT, and ASA. Therefore, excessive SeNPs resulted in the increase of lipid peroxidation and radicals, and the ability of Se-containing enzymes to scavenge radicals reached saturation. Consequently, the total antioxidant capacity of the body declined significantly at the nonlethal level. Shi and Spallholz found that although Se can scavenge oxygen radicals, there is too much Se in cells and the production of superoxide anion (O_2_^−^) in cells will be increased due to excessive oxidation of reduced GSH [35]. Bai et al. fed mice for 14 days with SeNPs synthesized by aqueous chitosan, and they found that SeNPs at doses of 0.5, 2, and 8 mg Se/kg BW increased TBARS (MDA equivalent), CAT, and GPX activities in the blood [36]. However, our results showed that SeNPs had no significant effect on the activity of GSH-Px in serum, with a decreasing tendency. The inconsistent results may be due to a different breed of experimental animals and the different preparation methods of SeNPs. In our study, CAT activities were increased dramatically at nonlethal levels, which may be related to the toxicity of Se and the high content of H_2_O_2_ in serum.

The liver is more sensitive to the toxicity from excessive SeNPs. The kidney of rats was more tolerant to excessive SeNPs than other organs. The antioxidant capacities of the liver, kidney, and heart were significantly impaired by the administration of SeNPs at a dose of 8 mg Se/kg BW. SeNPs impaired the antioxidant capacity of the liver and the heart at a dose of 4 mg Se/kg BW with varying degrees. SeNPs at a dose of 2 mg Se/kg BW decreased the T-AOC of the liver but did not affect kidney and heart T-AOC. The antioxidant capacity in the kidney was influenced by SeNPs inconsistently. SOD activity was increased, while TrxR activity was decreased. Zhang et al. reported that oral administration of 2 or 4 mg Se/kg BW SeNPs per day for 15 consecutive days increased CAT and GPX activities and decreased SOD and GSH activities in mice liver [37]. The results obtained in our experiment were highly consistent with their study. The changes of Se-containing enzymes in different organic tissues exhibited organizational specificity. It has been reported that the selenoprotein content in brain tissue, which could be affected by dietary Se supplementation, is less sensitive than other organic tissues [38]. In our experiment, inconsistent changes of Se-containing enzymes and antioxidant capacity indexes were found in different organic tissues with the same level of SeNP administration.

GPX1 is a major intracellular antioxidant enzyme, which decomposes hydrogen peroxide and some hydroperoxides (except phospholipid hydroperoxide); protects cells from oxidative stress; and prevents DNA damage, lipid peroxidation, and protein degradation [34]. The most basic function of GPX4 is to exert antioxidation in the body. In mammals, only glutathione peroxidase can directly degrade phospholipid hydroperoxide and play a role in apoptosis [39, 40].

Our results revealed that SeNPs have a significant effect on the mRNA expression of two Se-containing enzymes in the liver, namely, GPX1 and GPX4. Under selenium deficiency, the stability of mRNA and the level of mRNA of GPX decreased [41]. The study of Sunde and Raines showed that only about 4% of the gene expression levels of all 24 selenoproteins in rats were changed significantly at toxic doses of Se (50 times the dietary requirements) [42]. The gene expression of selenoprotein is regulated by numerous factors and multilevel regulation systems, and the expression of selenoprotein is tissue specific. The mechanism underneath excessive SeNPs on GPX gene expression needs to be further studied.

With TEM observation on the liver, we found that 8 mg Se/kg BW SeNPs caused visible damage to liver cells, and the injured cells had the characteristics of apoptosis. To further verify the effect of a high dose SeNPs on the antioxidant capacity of the liver and the mechanism of liver damage, antioxidation and cytotoxicity experiments in BRL cells were carried out.

In the study of BRL cells, we found that SeNP treatment below 12 *μ*M can promote the antioxidant capacity of cells, while SeNP treatment over 24 *μ*M damaged the antioxidant capacity of cells. The results indicated that SeNPs behave as an antioxidant at a lower dose and stimulate the activity of antioxidant enzymes in BRL cells, while in the case of a high dose, SeNPs turn into a prooxidizer. A high dose of SeNPs causes a large amount of accumulation of radicals in cells, which leads to changes in the activity of related enzymes, increasing intracellular peroxidation products and decreasing antioxidant capacity.

Many researchers suggested that SeNPs have an anticancer ability and better selectivity of various types of neoplastic cells with lower toxicity on normal cells [3]. However, exposure to SeNPs may pose more severe threats to Medaka compared to selenite due to Se accumulation in the liver [16]. In our study, cell viability was decreased to 87.76% and 51.37%, respectively, when treated with 24 *μ*M and 48 *μ*M SeNPs for 24 h. The study of Jiang et al. showed that GLP-SeNPs (SeNP surface decorator with *Gracilaria lemaneiformis* polysaccharide) exhibited broad-spectrum growth inhibition on typical malignant tumor cells, with IC50 values ranging from 9.1 ± 1.53 *μ*M (U87) to 27.6 ± 3.13 *μ*M (C6); the toxicity of GLP-SeNPs toward L02 cell IC50 value at 95.6 ± 7.68 *μ*M was much lower than those of cancer cells [43]. The results demonstrated that SeNPs possessed high selectivity between cancer and normal cells, and our results showed that SeNPs have cytotoxicity toward normal liver cells when treated with over 24 *μ*M SeNPs. Feng et al. also reported that SeNPs@Lys (Lys-modified SeNPs) displayed growth inhibition against MCF-7, Hela, and HepG2 cells with IC50 values ranging from 5 to 9.6 *μ*M. SeNPs@Lys has demonstrated lower cytotoxicity toward normal human kidney cells (HK-2) with IC50 values of 45.5 *μ*M, which was much higher than those of MCF-7, Hela, and Hep G2 cells [44]. The results of the present study showed that SeNPs have a toxic effect on hepatocytes when the concentration is higher than 24 *μ*M. SeNPs can be a potential application agent in cancer treatment only with appropriate dosage.

Several mechanisms have been proposed to illustrate the anticancer activity of Se, which includes cell apoptosis, restraint of cell multiplication, regulation of redox state, detoxification of cancer-causing agents, stimulation of the immune system, and inhibition of angiogenesis [45, 46]. Among these potential systems of anticancer activity of Se, apoptosis gets the most consideration and has been proposed to be fundamental for tumor chemoprevention by selenocompounds [47]. Apoptosis induced by SeNPs is described in various types of neoplastic cells [15, 16, 48]. Therefore, in our study, flow cytometry was used to verify whether apoptosis was involved in decreased cell viability induced by SeNPs. The results obtained indicated that decreased cell viability induced by SeNPs is mainly caused by apoptosis, rather than cell necrosis.

Extrinsic and intrinsic signaling pathways are the two major pathways leading to apoptosis. In both pathways, signaling results in the activation of a family of cysteine proteases, named caspases, which are the central regulators of apoptosis [16]. The caspase enzyme is mainly realized through the mitochondrial/Cytc pathway, the death receptor pathway, and the endoplasmic reticulum pathway. As a critical protease, Caspase-9 participates in intrinsic apoptosis signal transduction [49], while Caspase-8 participates in extrinsic apoptosis signal transduction and Caspase-3 is a crucial factor in the execution of apoptosis [50]. In our study, the activities of Caspase-3 and Caspase-8 were significantly increased, whereas no significant change was observed for Caspase-9 activity. A similar result was reported by Wu et al. [16]. However, Huang et al.'s research showed that Caspases-3, Caspase-8, and Caspase-9 were all triggered by SeNPs in a dose-dependent manner in MCF-7 cells [14]. Sun et al. also reported that Ru-SeNPs induced cells to undergo apoptosis and that apoptosis can be initiated through two separate pathways, intrinsic and extrinsic [51]. Therefore, different types of cells and different forms of SeNPs may have different approaches to induce apoptosis. Overall, the present results indicated that a high dose of SeNPs could impair the antioxidant capacity of cells and decrease cell viability through an extrinsic apoptosis pathway; however, the specific extrinsic pathway needs to be studied further.

Finally, it could be questioned whether SeNPs could be putatively used against specific pathologies. We could not arrive at an answer to this so far because the current study was designed to clarify the safety profile of SeNPs. However, a large number of studies have shown that SeNPs have valuable anticancer activity at high doses [47]. The anticancer mechanism of SeNPs is generally through the induction of excessive production of ROS in cancer cells, while excessive ROS can arrest the cell cycle [14], activate apoptosis signaling pathways, and finally lead to cancer cell apoptosis [52, 53]. The results of those studies and our present study suggest that SeNPs have the potential to be used as an anticancer drug, and it is worthwhile to carry out SeNP experiments in animal cancer models and even in cancer patients in future studies.

## 5. Conclusion

SeNP administration at the dose of 8.0 mg Se/kg BW significantly damaged the antioxidant capacity in serum, liver, kidney, and heart of rats, and liver is the most sensitive to the toxicity of pharmacological SeNPs. An in vitro study of BRL cells showed that SeNP treatment over 24 *μ*M impaired the antioxidant capacity of cells and led to cytotoxicity. SeNPs could induce cell death through apoptosis, and extrinsic pathways contribute to SeNP-induced apoptosis in BRL cells. And SeNPs have the potential to be used as a chemotherapeutic agent, especially for cancer treatment in the future.

## Figures and Tables

**Table 1:** Sequences of primers designed for RT-qPCR.

Gene	Forward sequences	Reverse sequences	GenBank accession # of mRNAs
GPX1	AGTCCACCGTGTATGCCTTCTCC	TCTCTTCATTCTTGCCATTCTCCTG	NM_030826
GPX4	GGAGGCAGGAGCCAGGAAGTAA	AGCCGTTCTTATCAATGAGAAACTTGG	NM_001039849
*β*-Actin	CGTTGACATCCGTAAAGACCTC	TAGGAGCCAGGGCAGTAATCT	NM_031144

GPX1: glutathione peroxidase 1; GPX4: glutathione peroxidase 4. *β*-Actin was used as an internal control to normalize target gene levels.

**Table 2:** Effect of SeNPs on antioxidant capacity in serum.

	Control	SeNPs		
		2 mg Se/kg BW	4 mg Se/kg BW	8 mg Se/kg BW
SOD (U/mL)	142.04 ± 6.48	153.39 ± 9.71	138.88 ± 10.78	128.11 ± 11.08
MDA (nmol/mL)	6.26 ± 0.54	6.1 ± 0.58	6.51 ± 0.62	7.79 ± 1.24^∗^
GSH-Px (U/mL)	1162.3 ± 70.28	1139 ± 108.74	1149.3 ± 129.43	1093.9 ± 85.19
CAT (U/mL)	0.90 ± 0.24	2.26 ± 0.63^∗∗^	2.95 ± 0.77^∗∗∗^	1.90 ± 0.67^∗^
T-AOC (U/mg)	2.72 ± 0.17	2.10 ± 0.41	2.22 ± 0.44	1.82 ± 0.26^∗^
T-GSH (mg/L)	45.20 ± 2.00	43.34 ± 4.84	42.77 ± 2.88	42.35 ± 2.2
GSSH (mg/L)	14.23 ± 0.95	14.63 ± 1.68	16.47 ± 0.99^∗^	20.03 ± 1.75^∗∗∗^
GSH (mg/L)	16.09 ± 0.93	20.11 ± 6.22	16.94 ± 1.64	13.51 ± 1.25^∗^
ASA (U/g of protein)	564.61 ± 5.96	579.93 ± 16.72	597.09 ± 17.07	426.1 ± 55.53^∗∗∗^
IHR (U/g of protein)	1331 ± 13.36	1342.8 ± 16.64	1316.2 ± 66.67	1295.5 ± 54.05
H_2_O_2_ (mmol/L)	28.18 ± 3.37	29.43 ± 5.58	40.09 ± 1.41^∗∗∗^	56.22 ± 4.73^∗∗∗^

Data are presented as the mean ± SD, *n* = 10. Control means the control treatment (without SeNP administration). ^∗^, ^∗∗^, and ^∗∗∗^ indicate significant difference as compared with the control treatment (*P* < 0.05, 0.01, and 0.001 respectively). SOD: superoxide dismutase; MDA: malondialdehyde; GSH-Px: glutathione peroxidase; CAT: catalase; T-AOC: total antioxidation capability; T-GSH: total glutathione; GSSH: oxidized glutathione; GSH: reduced glutathione; ASA: the abilities of antisuperoxide anion; IHR: inhibition of hydroxy radical.

**Table 3:** Effect of SeNPs on antioxidant capacity in BRL cells.

	Control	SeNPs					
		0.1 *μ*M	0.5 *μ*M	1 *μ*M	12 *μ*M	24 *μ*M	48 *μ*M
GSH-Px U/mL	50.76 ± 2.89	57.38 ± 1.43^∗∗^	67.37 ± 1.3^∗∗∗^	67.75 ± 2.25^∗∗∗^	50.29 ± 4.03	43.17 ± 3.25^∗∗^	38.07 ± 1.75^∗∗∗^
CAT U/mL	2.79 ± 0.10	2.82 ± 0.07	2.61 ± 0.23	2.80 ± 0.06	3.34 ± 0.21^∗∗^	4.00 ± 0.13^∗∗∗^	4.59 ± 0.26^∗∗∗^
SOD U/mL	16.49 ± 2.61	15.9 ± 1.81	16.4 ± 2.25	20.86 ± 1.51^∗^	21.44 ± 1.57^∗^	27.67 ± 2.62^∗∗∗^	7.89 ± 1.38^∗∗∗^
MDA nmol/mL	2.13 ± 0.39	0.97 ± 0.54	1.31 ± 0.25	1.54 ± 1.19	1.94 ± 0.61	5.83 ± 0.46^∗∗∗^	8.25 ± 0.86^∗∗∗^
T-AOC U/mg	0.43 ± 0.06	0.46 ± 0.07	0.6 ± 0.05∗	0.83 ± 0.16^∗∗∗^	0.74 ± 0.07^∗∗∗^	0.25 ± 0.05^∗^	0.16 ± 0.03^∗∗^

Antioxidant capacity in BRL cells treated with different concentrations (ranging from 0.1 to 48 *μ*M) of SeNPs for 24 h. Data are presented as the mean ± SD, *n* = 5. Control means the control treatment (the complete medium with 0 *μ*M SeNPs). ^∗^, ^∗∗^, and ^∗∗∗^ indicate significant difference as compared with the control (*P* < 0.05, 0.01, and 0.001 respectively). GSH-Px: glutathione peroxidase; CAT: catalase; MDA: malondialdehyde; SOD: superoxide dismutase; T-AOC: total antioxidation capability.

## Data Availability

The data used to support the findings of this study are available from the corresponding author upon request.
